# Explainable artificial intelligence-based prediction of poor neurological outcome from head computed tomography in the immediate post-resuscitation phase

**DOI:** 10.1038/s41598-023-32899-5

**Published:** 2023-04-08

**Authors:** Yasuyuki Kawai, Yohei Kogeichi, Koji Yamamoto, Keita Miyazaki, Hideki Asai, Hidetada Fukushima

**Affiliations:** Department of Emergency and Critical Care Medicine, Nara Medical University, 840 Shijo-cho, Kashihara, Nara 634-8522 Japan

**Keywords:** Outcomes research, Neurological disorders, Brain imaging

## Abstract

Predicting poor neurological outcomes after resuscitation is important for planning treatment strategies. We constructed an explainable artificial intelligence-based prognostic model using head computed tomography (CT) scans taken immediately within 3 h of resuscitation from cardiac arrest and compared its predictive accuracy with that of previous methods using gray-to-white matter ratio (GWR). We included 321 consecutive patients admitted to our institution after resuscitation for out-of-hospital cardiopulmonary arrest with circulation resumption over 6 years. A machine learning model using head CT images with transfer learning was used to predict the neurological outcomes at 1 month. These predictions were compared with the predictions of GWR for multiple regions of interest in head CT using receiver operating characteristic (ROC)-area under curve (AUC) and precision recall (PR)-AUC. The regions of focus were visualized using a heatmap. Both methods had similar ROC-AUCs, but the machine learning model had a higher PR-AUC (0.73 vs. 0.58). The machine learning-focused area of interest for classification was the boundary between gray and white matter, which overlapped with the area of focus when diagnosing hypoxic– ischemic brain injury. The machine learning model for predicting poor outcomes had superior accuracy to conventional methods and could help optimize treatment.

## Introduction

With the poor prognosis of out-of-hospital cardiopulmonary arrest^1^, predicting neurological outcomes after resuscitation is important for appropriate resource utilization, identifying whether patients will benefit from interventions, and early identification of those at risk of brain death. Therefore, several factors that are quantifiable at the bedside, including clinical findings, biomarkers (such as neuron-specific enolase), and neuro-electrophysiological test data (electroencephalography and somatosensory evoked potential), have been evaluated as predictors of neurological prognosis^2^. However, individual tests that are performed immediately after resuscitation and over 72 h require multimodal prognostication^3,4^, and the results of the tests may be influenced by post-resuscitation sedation.

In contrast, imaging has the advantage of no interference from sedative use; furthermore, the diagnosis of hypoxic–ischemic brain injury (HIBI) on head computed tomography (CT) images obtained immediately after resuscitation is a specific predictor of poor neurological outcomes^5–7^. However, the radiodiagnostic criteria for HIBI remain unclear, and the evaluation involving the use of the gray-to-white matter ratio (GWR) from CT images, which is the most commonly used evaluation method for HIBI, can be susceptible to low reproducibility, since the regions of interest (ROIs) in the brain are selected manually^2^.

In recent years, machine learning has significantly improved the accuracy of image classification and is being utilized increasingly in medicine^8–10^. Although only a few studies have investigated ischemic changes, such as post-resuscitation cerebral infarction, this technology potentially has high accuracy for identifying subtle changes that evade the human visual detection threshold^8^. However, a previous study^8^ used a model to predict HIBI using head CT-based results that appeared late in the time series, thus hindering the elimination of data leakage, which can influence the accuracy.

Therefore, in this study, we hypothesized that a machine learning model can enable the identification of poor prognostic features in head CT scans immediately after resuscitation that cannot be detected by humans even with the use of clear diagnostic criteria. The primary objective of this study was to determine the accuracy of the predictions of neurological outcomes using machine learning models by comparing them with the predictions of the existing GWR method based on CT images of the head immediately after resuscitation. The secondary objective was to provide a heatmap visualization of the areas on which the machine learning model based its classification to provide information for physicians to understand the basis of the model’s decision-making process.

## Results

### Participant characteristics

**Figure 1:**
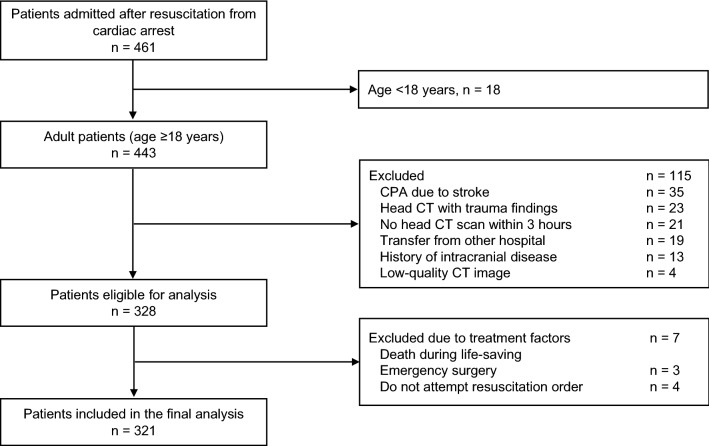
Flowchart of participant selection in the study. *CPA* cardiopulmonary arrest, *CT* computed tomography.

During the observation period, 443 adult patients (age ≥ 18 years) were admitted to the hospital after resumption of circulation. Head CT was performed in 95% of these patients, and 321 patients were included in this study (Fig. 1). The baseline characteristics of the study population are shown in Table 1. The median age of the cohort was 69 years (interquartile range: 57–78 years), and 213 (66.6%) patients were men. Head CT was performed under return of spontaneous circulation (ROSC) in 87.2% patients. For the remaining patients, it was performed under veno-arterial extracorporeal membrane oxygenation-assisted circulation. Since 67 (20.9%) patients had good neurological outcomes, the dataset was imbalanced in terms of outcomes. Of these, five patients who died within 1 month but were classified as having a good prognosis based on their condition after CT imaging, according to this study’s classification, were included. The durations of cardiac arrest in these five patients were 19, 23, 37, 19, and 63 min. The causes of death were cardiogenic and respiratory in three and two patients, respectively. Cardiogenic death occurred in one patient on the same day when cardiac arrest had occurred, whereas the other four patients survived for more than 14 days.

**Table 1:** Participant characteristics.

Variable	Overall	Good outcome	Poor outcome	*P*-value
	n = 321	n = 67	n = 254	
Age, years	69 [57, 78]	65 [54, 73]	70 [58, 79]	0.009
Male, n	213 (66.6)	45 (68.2)	168 (66.1)	0.88
Witness, n	222 (69.2)	54 (80.6)	168 (66.1)	0.03
Bystander CPR, n	171 (53.3)	44 (65.7)	127 (50.0)	0.03
Cardiac cause, n	173 (53.9)	48 (71.6)	125 (49.2)	0.001
Shockable rhythm, n	82 (25.5)	35 (52.2)	47 (18.5)	< 0.001
Call to return of circulation interval, min	38 [24, 55]	17 [9, 27]	43 [30, 61]	< 0.001
CT under ROSC*, n	280 (87.2)	62 (92.5)	218 (85.8)	0.22
Return of circulation to CT interval, min	36 [26, 51]	42 [31, 69]	34 [25, 49]	0.001
Call to CT interval, min	79 [63, 98]	69 [53, 91]	82 [66, 100]	< 0.001
TTM, n	107 (33.3)	24 (35.8)	83 (32.7)	0.66
CPC 1 or 2 at 1 month, n	62 (19.3)			
Death within 1 month, n	208 (64.8)	5 (7.5)	203 (79.9)	< 0.001

*CPR* cardiopulmonary resuscitation, *CT* computed tomography, *ROSC* return of spontaneous circulation, *VA-ECMO* veno-arterial extracorporeal membrane oxygenation, *TTM* target temperature management, *CPC* cerebral performance category, *IQR* interquartile range.

*The remainder of patients underwent CT under extracorporeal support with VA-ECMO.

From the entire dataset, 257 cases were used to construct the model, and 64 cases were used to validate the model. Training was stopped at 35 epochs of decreasing accuracy in the validation data. A summary of the GWR measurements is presented in Table 2. The Spearman’s rank correlation coefficient of the correlation between the two measurements was higher than 0.8. Patients predicted to have a poor outcome with a cut-off value of 1.2 had a basal ganglia GWR (GWR-BG) of 76, a cerebral GWR (GWR-CE) of 50, and an average GWR (GWR-AV) of 44.

**Table 2:** Gray-to-white matter ratio measured based on previously reported methods.

			Average of two measurements	Spearman's rank correlation coefficient	Neurological outcome		*P-*value
					Good	Poor	
			n = 321		n = 67	n = 254	
Basal ganglia	Gray matter	CN	32.5 [30.8, 34.3]	0.89	34.8 [33.3, 36.3]	32.0 [30.5, 33.5]	< 0.001
		PU	33.0 [31.3, 34.8]	0.84	35.3 [33.8, 36.5]	32.3 [30.8, 34.3]	< 0.001
		THL	32.0 [30.3, 33.8]	0.85	33.8 [32.5, 35.5]	31.5 [29.8, 33.3]	< 0.001
	White matter	CC	26.3 [25.0, 27.8]	0.81	26.8 [25.5, 28.3]	26.3 [25.0, 27.5]	0.09
		PLIC	26.0 [24.8, 27.3]	0.80	26.5 [25.3, 27.7]	26.0 [24.8, 27.3]	0.05
Cerebrum	Gray matter	MC1	31.5 [29.8, 34.5]	0.83	34.0 [31.4, 36.7]	31.3 [28.9, 33.7]	< 0.001
		MC2	31.0 [27.8, 34.0]	0.88	32.5 [30.3, 35.9]	30.3 [27.8, 33.3]	< 0.001
	White matter	MWM1	24.5 [23.3, 26.0]	0.77	25.3 [23.4, 27.2]	24.3 [23.0, 25.8]	0.01
		MWM2	25.0 [22.8, 27.0]	0.86	25.5 [23.0, 27.8]	24.8 [22.8, 27.0]	0.08
GWR	Basal ganglia	GWR-BG	1.25 [1.20, 1.30]	0.76	1.31 [1.27, 1.36]	1.23 [1.19, 1.28]	< 0.001
	Cerebrum	GWR-CE	1.26 [1.21, 1.31]	0.55	1.32 [1.27, 1.36]	1.25 [1.21, 1.30]	< 0.001
	Average	GWR-AV	1.25 [1.21, 1.30]	0.71	1.31 [1.29, 1.35]	1.24 [1.21, 1.28]	< 0.001

Median (interquartile range) GWR for survivors and non-survivors.

*CN* caudate nucleus, *PU* putamen, *THL* thalamus, *CC* corpus callosum, *PLIC* posterior limb of the internal capsule, *MC* medial cortex, *MWM* medial white matter, *GWR* gray-to-white matter ratio, *BG* basal ganglia, *CE* cerebrum, *AV* average of BG and CE.

**Figure 2:**
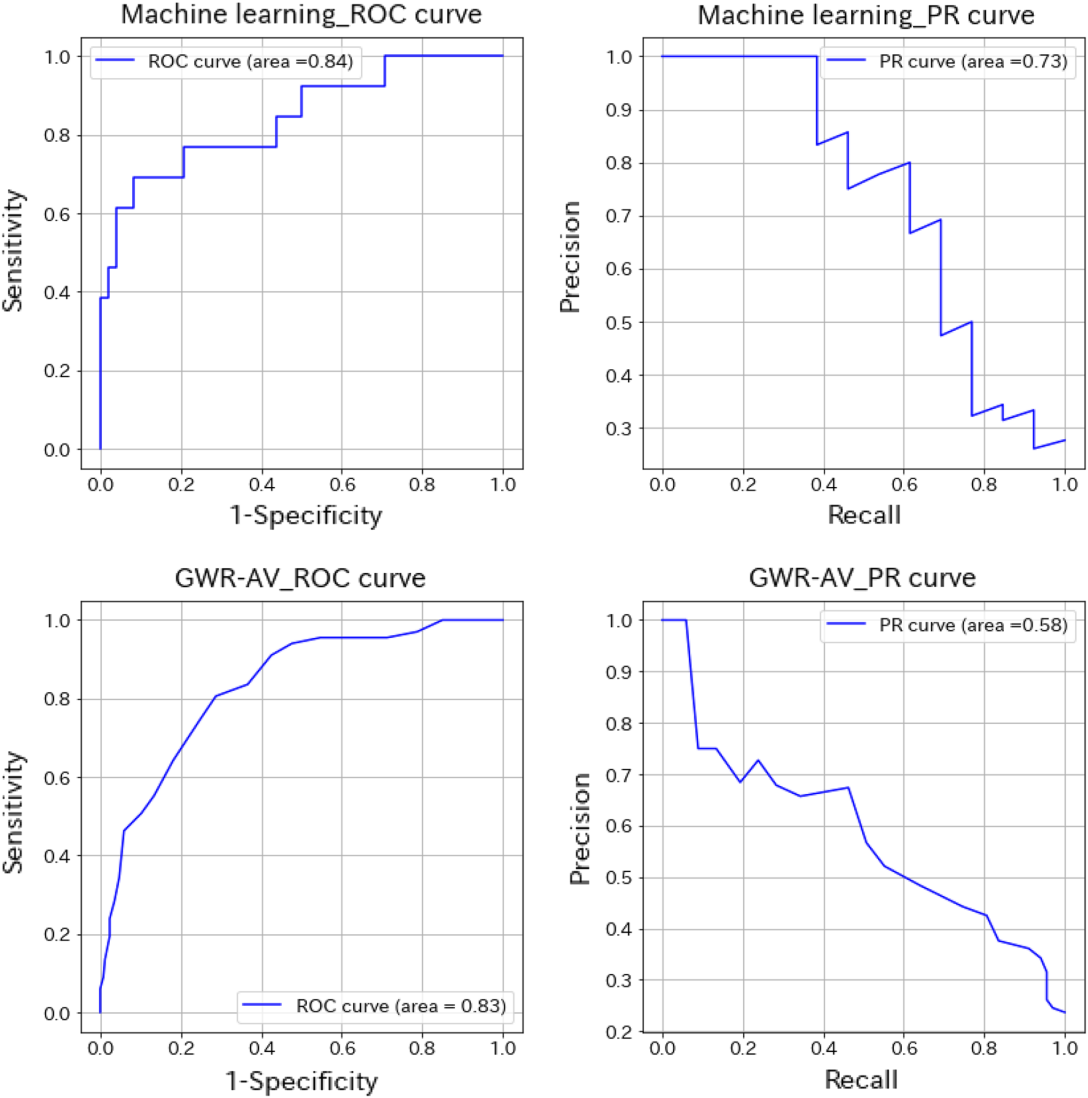
Machine learning model and GWR method for predicting poor outcomes. *ROC curve* receiver operating characteristic curve, *PR curve* precision recall curve, *GWR* gray-to-white matter ratio.

The machine learning prediction of poor outcomes was determined as a receiver operating characteristic (ROC)-area under the curve (AUC) of 0.84 and a precision recall (PR)-AUC of 0.73. The positive predictive value for a poor outcome was 0.92. For predicting poor outcomes with GWR using the same images, the GWR-AV of the basal ganglia and cerebrum had the best performance, with an ROC-AUC of 0.83 and a PR-AUC of 0.58. The positive predictive value was 0.24 (cut-off value: 1.2; Fig. 2).

To enable the comparison of our results with those of other studies, the data classified by cerebral performance category (CPC), without any modifications, at 1 month was subjected to the same analysis as a sensitivity analysis. Training was stopped at 30 epochs, and the results showed a similar trend to that in the unrevised dataset. Machine learning predicted poor outcomes with an ROC-AUC of 0.85 and a PR-AUC of 0.71. The positive predictive value for a poor outcome was 0.88. For predicting poor outcomes with GWR using the same images, the GWR-AV had the best performance, with an ROC-AUC of 0.82 and PR-AUC of 0.56 (positive predictive value: 0.24, cut-off value: 1.2).

**Figure 3:**
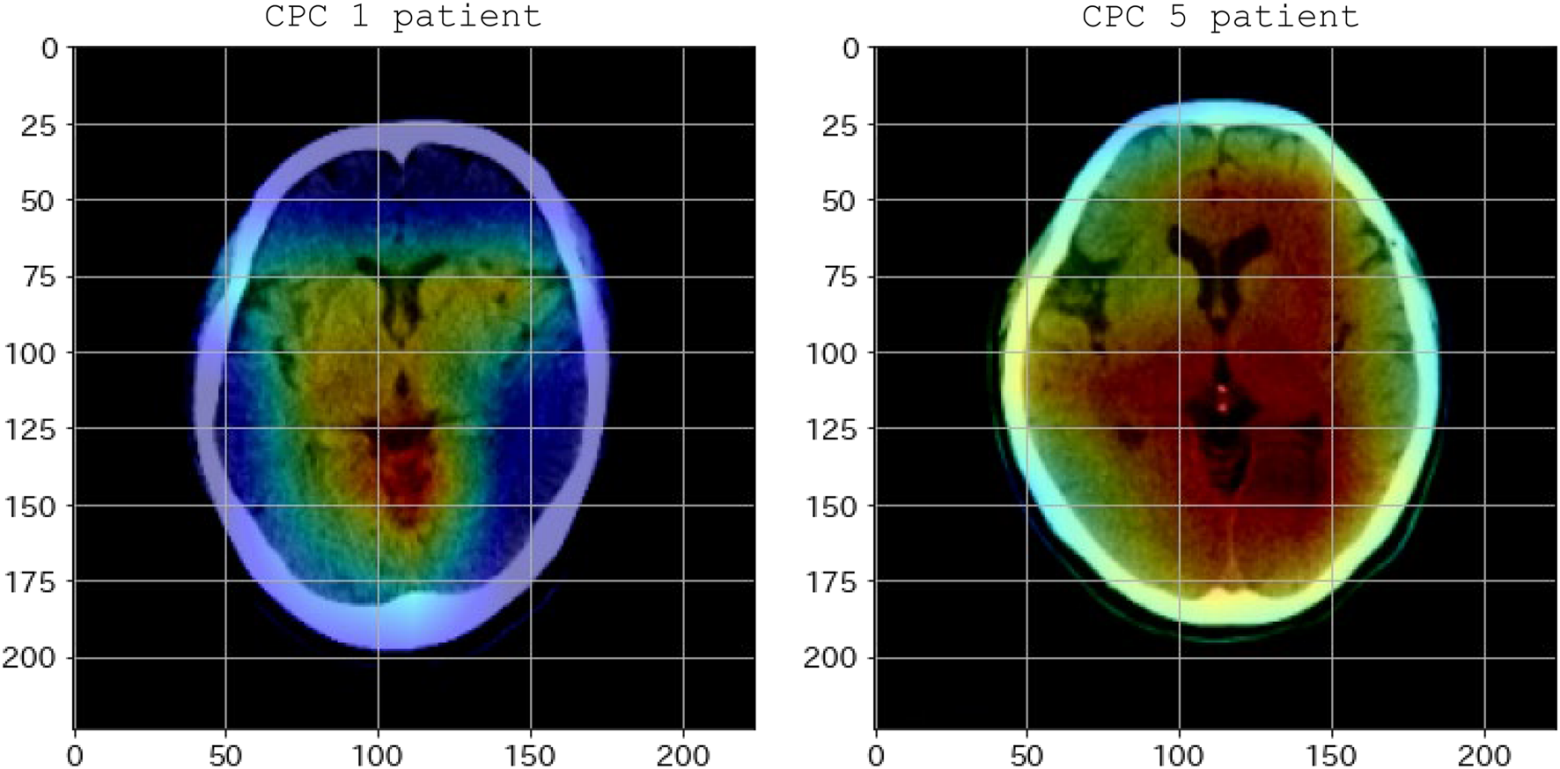
Heatmap of good and poor neurological outcomes generated by Grad-CAM. The red regions correspond to high scores for the class. *CPC* cerebral performance category.

Heatmap visualization with gradient-weighted class activation mapping (Grad-CAM) showed that the prediction of good neurological outcomes was based on the basal ganglia. In contrast, poor neurological outcomes were strongly influenced by the wide area of the cerebrum, excluding the brain surface (Fig. 3).

## Discussion

The machine learning model was able to predict the neurological outcomes at 1 month with high accuracy using only the features of head CT that were obtained immediately after resuscitation. The accuracy of machine learning predictions was comparable to that of the GWR-based predictions in terms of the ROC-AUC and higher than that of the GWR-based predictions in terms of the PR-AUC. To the best of our knowledge, this is the first study to directly predict neurological outcomes at 1 month by applying an explainable machine learning model to only head CT images that were obtained immediately after resuscitation.

HIBI on post-resuscitation head CT is considered to be a finding suggestive of poor neurological outcomes, but no accurate method of diagnosis is currently available. HIBI is recognized on post-resuscitation head CT as decreased differentiation of gray and white matter and effacement of the cortical sulci^11^. However, the findings indicating HIBI on early head CT are highly variable. There are no guidelines for a clear, consistent definition of HIBI^12^, and there is a low interrater agreement rate^13^, although this depends on the expertise of the radiologists^13,14^. Therefore, we selected the GWR, which is a quantitative measure of HIBI, for comparison with machine learning. In this study, the consistency between two the measurements obtained with the GWR method was good.

The machine learning and GWR-based predictions were comparable in terms of the ROC-AUC, but false positives could not be eliminated by the GWR method. A possible reason for the difficulty in eliminating false-positive patients may be that this study analyzed imbalanced data, which makes it difficult to correctly predict the classifications^15,16^. Although there is no clear definition of imbalanced data, a label deviation of approximately 8:2 generally hampers analysis. According to our institution’s policy, head CT was performed for almost all patients after resuscitation, which may have contributed to the similar imbalance ratio to the predicted post-resuscitation neurological outcome ratio. We adjusted label weights to improve the accuracy of our machine learning model and found that the machine learning model in this study had a sensitivity of 0.4 when false positives were set to 0%, meaning its predictions are as accurate or more accurate than the predictions of the GWR method. As the elimination of false positives is important for predicting poor outcomes after resuscitation, we believe that machine learning may be superior to GWR for imbalanced datasets, such as that used in this study.

Machine learning models are expected to be highly accurate; however, the process of obtaining the results is complex, and the basis for decisions is difficult to understand. Grad-CAM facilitates human interpretation using a heatmap to visualize the areas that are considered important when machine learning determines the results. The areas used to predict poor outcomes in the current study seem to correspond to the border between gray and white matter, an area that humans focus on when making decisions, although there are no clear criteria.

This study has several limitations. First, the study was conducted at a single institution, which limited its generalizability, and used a small dataset for machine learning. To eliminate the influence of clinical background on the outcome, the data collection period was limited, making it difficult to recruit a large number of eligible patients. Therefore, we used transfer learning, which uses pre-trained knowledge on large amounts of data, and data augmentation. Augmentation is effective in preventing overlearning by increasing the variety of data, although there is a risk that the same features will be augmented, thereby causing overlearning on the dataset. Therefore, it is necessary to validate this model using a large amount of external data to evaluate the generalization performance of the results of this study. In addition, because we limited the time of the head CT to within 3 h after resuscitation from cardiac arrest, which was the actual time at our institution, we were unable to evaluate the images beyond that time.

Second, the heatmap created by Grad-CAM visualizes the areas focused on as the basis for classification. However, this does not explain the clinical significance of these areas. In the cases of patients with poor outcome, the important regions are spread over a wide part of the cerebrum, excluding the cerebral surface, and the focus appears to be on regions where there are many gray and white matter boundaries; however, this is a human interpretation. Focusing on the regions the machine learning models consider important for classification may include features that are not recognized by humans and may be useful for more accurate prognosis prediction in the future.

Third, our unique and original neurological outcome classification scheme was used to classify the outcomes in this study. For supervised machine learning, it is important to correctly label the data. In particular, as this study aimed to predict outcomes using only head CT information obtained immediately after resuscitation, it was considered necessary to use a modified classification for the deaths of patients presumed to be caused by non-neurogenic causes after head CT (originally classified as CPC5) showed good neurological outcomes. A sensitivity analysis using a dataset without modifying the CPC yielded similar results, but the modified classification may have led to biased results.

Finally, although withdrawal of life-sustaining therapy is not performed at our institution, we were unable to confirm that the outcome was not influenced by a self-fulfilling prophecy bias, as this was a retrospective study of head CT findings.

## Conclusion

This study showed that a machine learning model for predicting poor outcomes using head CT images immediately after resuscitation may perform as well as or better than previous methods. Verification studies using a large amount of highly diverse data should be conducted in the future to confirm the requirements for enhancing the accuracy of these models for clinical use.

## Methods

### Study design and population

This single-center, retrospective, observational study was conducted using data from the medical records of patients with out-of-hospital cardiopulmonary arrest who were transported to the Nara Medical University Advanced Critical Care and Emergency Centre. This study was approved by the institutional ethics committee of Nara Medical University (No. 3131). Since this was an observational study, the need for written informed consent was waived by the ethics committee of Nara Medical University. This study report follows the TRIPOD guidelines^17^. All methods in our study were performed in accordance with the tenets of the Declaration of Helsinki.

Between April 2015 and March 2021, consecutive patients with out-of-hospital cardiopulmonary arrest who were admitted and treated for ROSC or extracorporeal circulation were included in this study. Patients who were under 18 years old; patients with head trauma, stroke, and previous intracranial disease, except lacunar infarction; patients transported from other hospitals; patients who did not undergo head CT imaging within 3 h after resuscitation (based on the CT imaging duration of previous studies^2^); patients with inadequate CT imaging coverage; patients who died during surgery for aortic rupture immediately after cardiopulmonary resuscitation; and patients who were withdrawn from life-sustaining therapy after ROSC at their or their family’s request were excluded. Withdrawal of life-sustaining treatment is not performed at our institution.

### Post-resuscitation care

According to our standardized protocol, the patients were managed with sedation, analgesia, and ventilation according to the resuscitation guidelines^18^. The exclusion criterion for target temperature management (TTM) was shock (systolic blood pressure < 90 mmHg) despite the use of vasopressors. Central body temperature was maintained at 33 °C for 24 h using the Arctic Sun® Temperature Management System (Bard, BD, Covington, GA, USA), followed by rewarming at a rate of 0.25 °C/h and maintained at 37 °C for additional 24 h. If TTM could not be performed, other treatment was performed in the similar manner.

### Data collection of participant characteristics

The following data were collected retrospectively from the electronic medical records: age, sex, witness, bystander, initial rhythm, cause of cardiac arrest, time from cardiac arrest to circulation resumption, time from circulation resumption to CT scan, and CPC at 1 month after resuscitation for inpatients and at outpatient follow-up for discharged patients.

### Original neurological outcome assessment

Current recommendations define poor neurological outcome as a CPC^19^ of 3–5^20,21^. Based on the minimum acceptable time of up to 1 month after resuscitation, the neurological outcome of a patient with a good neurological outcome on head CT immediately after resuscitation who died within 1 month was classified as CPC 5, which indicates poor neurological outcome. Accurate labels are indispensable for training the machine learning models on various data features. As this study aimed to predict the neurological outcomes from head CT imaging data, neurological outcome information must be reflected in the training data (i.e., the head CT). The criteria for the presumption of CPC 1 or 2 are that the patient is awake, able to communicate and perform the indicated actions without disability, and has no evidence of paralysis in the extremities. To minimize the risk of misclassification in the present study, the patients classified as CPC 1 or 2 after CT imaging who died within 1 month of admission but whose deaths were not attributable to intracranial disease were still classified as CPC 1 or 2. Since these patients were presumed to have good neurological outcomes after CT was performed, we did not consider it appropriate to exclude them a priori from the study, which aimed to predict neurological outcomes based on head CT imaging.

### CT protocol and conditions for image collection

All CT images were acquired with a 64-row helical CT system (Optima CT660; GE Healthcare, Chicago, IL). The scan settings were as follows: 120 kVp; auto mA; rotation time, 0.5 s; helical pitch, 0.531; noise index, 3.0; and image noise, SD10. In post-resuscitation CT imaging, decreasing the examination time is a priority. Therefore, in this study, reconstructed images with an orbitomeatal baseline were used to reduce variations in the imaging conditions. The CT images used for machine learning were of slices at the levels of Monroe’s foramen and the pineal gland that are used in GWR-based studies. Images were acquired as portable network graphics with a size of 1 × 256 × 256 under a window level of 40 Hounsfield unit (HU) and width of 80 HU.

### Machine learning model

**Figure 4:**
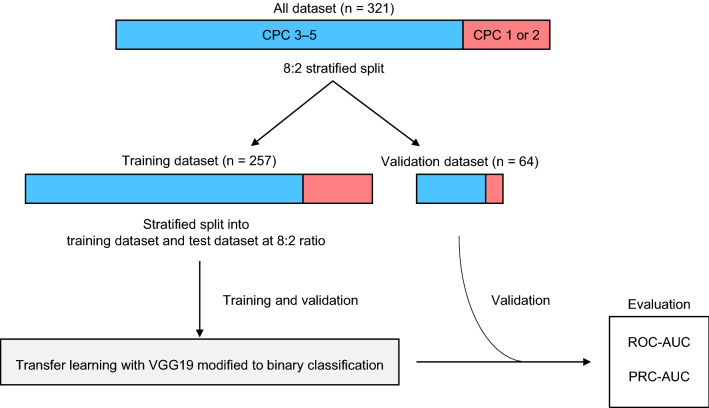
Overview of the training and validation sets used in this study. *CPC* cerebral performance category, *ROC_AUC* receiver operating characteristic area under the curve, *PRC_AUC* precision recall curve area under the curve.

The prepared image dataset was stratified and divided into training and validation datasets at the commonly used ratio of 8:2. Then, the training dataset was stratified and divided into training dataset and test dataset at a ratio of 8:2 and used to construct the model. The validation data, which was not used for training, was used to validate the model. The VGG19^22^ machine learning model was used; it is a 19-layer convolutional neural network, with transfer learning for applying parameters that were obtained from training with 1 million images (Fig. 4). Transfer learning is a method of transferring learning on large amounts of high-quality data to create highly accurate models for a small dataset. Although models with better predictive accuracy are available, VGG19 was used in the present study because of the high accuracy achieved by this relatively simple model in a previous study on post-resuscitation head CT^8^.

Image data acquired at a size of 256 × 256 were cropped in the center and resized to 224 × 224, followed by normalization. As the number of data was small, the data were adapted to image augmentation using transformations. Image augmentation is the creation of a new training sample from an existing image by slightly changing the original images. In this study, we induced random changes in the training data by adjusting sharpness, rotation, and erasing. As the dataset comprised imbalanced data that was biased in class, adjustments were made using weights. A grid search was used to tune the hyperparameters. To avoid overfitting the model on the current dataset, the number of epochs was determined by “earlystopping”, which stops training when the accuracy of the validation data decreases.

We further explored the areas of focus using the Grad-CAM technique to generate a “visual explanation” for the class decision of the model^13^. The Grad-CAM technique uses gradients that flow into the final convolution layer to produce a coarse localization map that highlights important regions in the image to predict the classes.

### Measurement of GWRs based on CT scans

The GWRs were measured for all patients in the study using a previously described method^5–7,23^. Briefly, head CT scans were retrospectively reviewed twice by an emergency physician blinded to patient outcomes. We measured the average HU of the circular ROI (10.0–15 mm^2^) on each side of the basal ganglia, centrum semiovale, and high cortical level. The caudate nucleus (CN), putamen (PU), posterior limb of internal capsule (PLIC), and corpus callosum (CC) were measured at the basal ganglia level, and the medial cortex (MC) and medial white matter (MW) were measured at the centrum semiovale level (MC1 and MW1) and high cortical level (MC2 and MW2), respectively. The relationship between the two measurements was evaluated using Spearman’s correlation coefficient, and the average of both measurements was used for subsequent evaluation. The GWRs were calculated according to previously reported equations as follows: GWR-BG = (CN + PU)/(PLIC + CC), GWR-CE = (MC1 + MC2)/(MW1 + MW2), and GWR-AV = (GWR-BG + GWR-CE)/2. In this study, we used a GWR cut-off value of 1.2, as mentioned in previous studies^5^.

### Statistical analysis

Continuous variables are expressed as median (interquartile range) and categorical variables as number of patients (percentages). The Mann–Whitney *U* and Fisher’s exact tests were used to compare continuous and categorical variables, respectively. Statistical significance was set at* P* < 0.05. The neurological outcome prediction performance of the methods was assessed by plotting the ROC curves and comparing the AUCs. As this study’s dataset comprised imbalanced data with labels at a ratio of approximately 8:2, a PR curve was drawn, and the AUCs were compared. ROC curves are frequently used to compare the performances of models since they are unaffected by the class proportions of the data. However, as our study cohort was considered to have a class imbalance (a small number of CPC 1/2), we had to consider the class bias. PR curves provide a good picture of the performance of a method when the ratio of classes in the test data is close to the ratio expected when the model is practically applied^24^. All analyses were performed using Python 3.8.5 (Python Software Foundation, Beaverton, OR).

## Data Availability

The data that support the findings of this study are available from the corresponding author, Y.Ka, on reasonable request.
